# Smoking Cessation and Benefits to Cardiovascular Health: A Review of Literature

**DOI:** 10.7759/cureus.35966

**Published:** 2023-03-09

**Authors:** Ovie Okorare, Endurance O Evbayekha, Olanrewaju K Adabale, Emmanuel Daniel, Daniel Ubokudum, Soremi A Olusiji, Akanimo U Antia

**Affiliations:** 1 Internal Medicine, Vassar Brothers Medical Center, Nuvance Health, New York City, USA; 2 Internal Medicine, St. Luke's Hospital, Chesterfield, USA; 3 Internal Medicine, East Carolina University, Greenville, USA; 4 Internal Medicine, Trinity Health Ann Arbor, Michigan, USA; 5 Internal Medicine, Thomas Hospital, Alabama, USA; 6 Internal Medicine, New York Medical College, Metropolitan Hospital Center, New York City, USA; 7 Medicine, Lincoln Medical and Mental Health Center, New York City, USA

**Keywords:** cigarette, cardiovascular, tobacco, nicotine, smoking

## Abstract

Tobacco smoking is a chief cause of preventable deaths worldwide, accounting for various cancers, cardiovascular and respiratory diseases. Tobacco smoking accounts for more than seven million deaths every year. Worldwide statistics show that about 1.1 billion active smokers exist; 80% live in low- and middle-income countries.

Nicotine is the addictive ingredient with the least harm compared to other active ingredients in tobacco, albeit not completely benign. Nicotine acts on the nicotinic cholinergic receptors (nAChRs) and produces the release of neurotransmitters. The mechanism by which it affects the cardiovascular system involves endothelial dysfunction by reducing nitrogen monoxide production, pro-thrombotic conditions, and activating inflammatory routes. These factors, along with the increased amounts of coronary atherosclerosis, have addictive adverse effects. Smoking has been shown to cause increased amounts of coronary atherosclerosis which may be responsible for the increased risk of hypertension, coronary heart disease, and atrial fibrillation, potentially contributing to the association of current smokers with a higher incidence of heart failure. This has led to worsened burdens and outcomes of cardiovascular disease among smokers.

Smoking cessation has been associated with a reduction in cardiovascular mortality. This ranges from the reduction in the incidence of hypertension, type 2 diabetes, and heart failure. As regards behavioral and mental health, smoking cessation reduces the risk of cardiovascular disease in people experiencing mental illness.

The prevalence of smoking continues to trend downward over the past couple of decades. Despite this downtrend, cigarette smoking is responsible for approximately half a million deaths per year in the United States and billions of dollars spent in healthcare. This buttresses the need to explore the various effects of smoking cessation on cardiovascular health and suggest ways to curb the disease burden.

## Introduction and background

Smoking is a prominent cause of preventable deaths worldwide, accounting for various cancers and cardiovascular and respiratory diseases. The smoking habit stems from the addiction to nicotine, a component of tobacco products. The release of neurotransmitters, including dopamine, in the brain creates a positive feedback mechanism that leads to smokers' dependence on tobacco [1].

Tobacco smoking accounts for more than seven million deaths every year. Worldwide statistics show that about 1.1 billion active smokers exist with 80% live in low- and middle-income countries. Nicotine is the addictive ingredient with the least harm compared to other active ingredients in tobacco, albeit not completely benign. Though global current smoking rates among adults have decreased from 23.5% to 20.7% between 2007 and 2015, this reduction was primarily due to the declining smoking rates in Northern and Western Europe, North America, and the Western Pacific regions, for which considerable measures were implemented to tackle tobacco smoking. Conversely, the smoking rate appears to be increasing in the Middle East and Africa. For example, in sub-Saharan Africa, tobacco consumption increased by 57% between 1990 and 2009 [2]. In general populations, the adverse effects of smoking on cardiovascular health have been demonstrated to be greater in women than in men. Compared with never-smokers, women who smoke have a 25% greater relative risk of developing coronary heart disease than men, independent of sex differences in the levels of other cardiovascular risk factors [3]. This review aims to investigate the motivation for smoking cessation and its benefits to cardiovascular health.

## Review

Methodology

Our search strategy and study selection are based on a defined set of inclusion and exclusion criteria.

Inclusion Criteria

**Figure 1:**
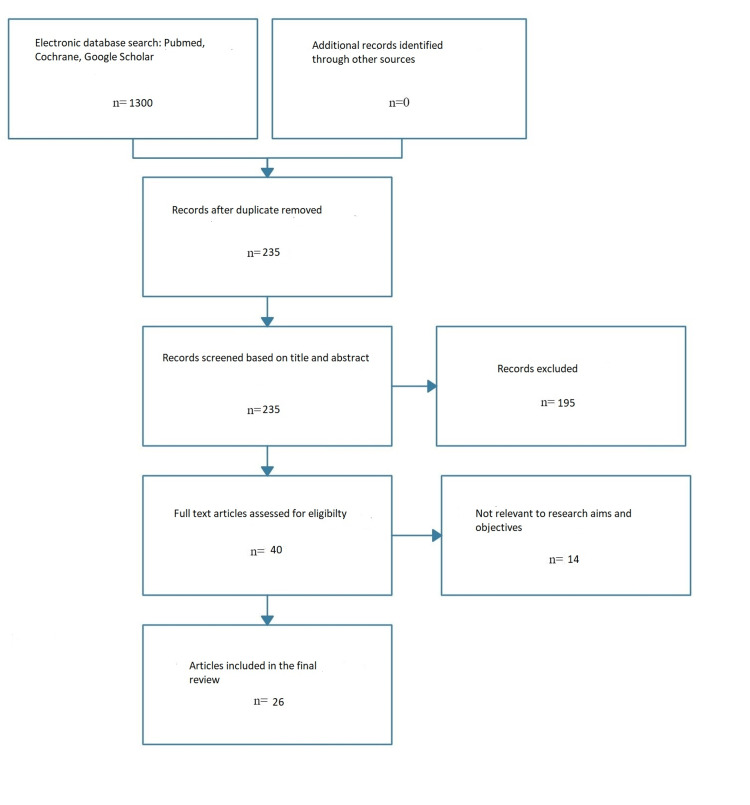
PRISMA flow chart detailing the systematic search

Studies that were published within the last 10 years. We conducted the search in PubMed, Google Scholar, and Cochrane. We included articles from the previous 10 years (2013 to 2023) and considered systematic reviews, meta-analyses, randomized control trials, and clinical trials. Keywords for the search included [(smoking) OR (cessation)] OR [(cardiovascular health) OR (guidelines)] in different combinations. We customized the searches according to the layout of each search engine and made every possible combination of the search keywords to generate the included articles. Our keyword combinations and search results generated 235 articles. We read the abstracts considering our objectives, inclusion, and exclusion criteria, then further narrowed them down to 40 full text articles and included 26 in our review as shown in Figure 1.

Exclusion Criteria

We excluded all studies published more than 10 years ago, case reports, case series, articles in languages other than English, and non-full-text articles.

Discussion

Smoking Epidemiology and Prevalence

Cigarette smoking is one of the commonest modifiable lifestyle risk factors. It is a significant contributor to preventable disease and a leading cause of death in the United States [4]. A meta-analysis of the prevalence of cigarette smoking among females revealed that cigarette smoking prevalence was high across all age groups, from adolescents to pregnant women, with the least prevalence among Asian women (14%) and the highest prevalence among the European female population (38%) [2].

The American Lung Association's analysis of the Center for Disease Control reported data which revealed that cigarette smoking had experienced a downward trend over the last 55 years, from 42.4% among adults in 1965 to 13.7% in 2018, and from 27.5% among youths in 1991, to 8.8% in 2017 [4,5]. The most recent data showed a decline in smoking from 20.9% among adults in 2005 to 12.5% in 2020 [6,7]. In this study, they defined current smokers as individuals who had a minimum of 100 cigarettes in their lifetime. They found that men were more likely than women to smoke cigarettes 14.1% vs. 11.0%. They also found that non-Hispanic American Indians/Alaska Native adults had the highest prevalence of cigarette smoking at 27.1%, followed closely by Blacks at 14.4%, Whites at 13.3%, Hispanics at 8.0%, and Asians at 8.0% [4].

Despite this downtrend, cigarette smoking is responsible for approximately half a million deaths per year in the United States and more than $600 billion lost in healthcare spending and lost productivity [6,7].

Pathophysiology of Smoking Addiction and Its Effect on Different Systems

Nicotine belongs to the amine poly-carbon group found in tobacco and tobacco products. It is an addictive agent that confers a much lower risk than other tobacco elements but is not completely benign. Tobacco smoke is rapidly absorbed into the bloodstream when inhaled. Inhaled nicotine escapes the first pass through intestinal and liver metabolism. Nicotine readily crosses the blood-brain barrier, promptly diffusing into the cerebral tissue; and this process is relatively fast between 2 and 8 seconds from time of inhalation [8].

It selectively binds to nicotinic cholinergic receptors (nAChRs) in the brain and other tissues and has a half-life of about two hours [8]. Imaging studies of the brain have demonstrated an acute increase in brain activity following ingestion of nicotine, especially within the thalamus, prefrontal cortex, and visual system, consistent with activating cortico-basal ganglia and thalamic brain circuits. Nicotine stimulates nAChRs, and releases neurotransmitters, predominantly dopamine, but also norepinephrine, acetylcholine, serotonin, gamma-aminobutyric acid, glutamate, and endorphins. These neurotransmitters cause various responses and behaviors after nicotine intake. When there is repeated exposure to nicotine, tolerance develops to some physiological effects of nicotine [8].

**Figure 2:**
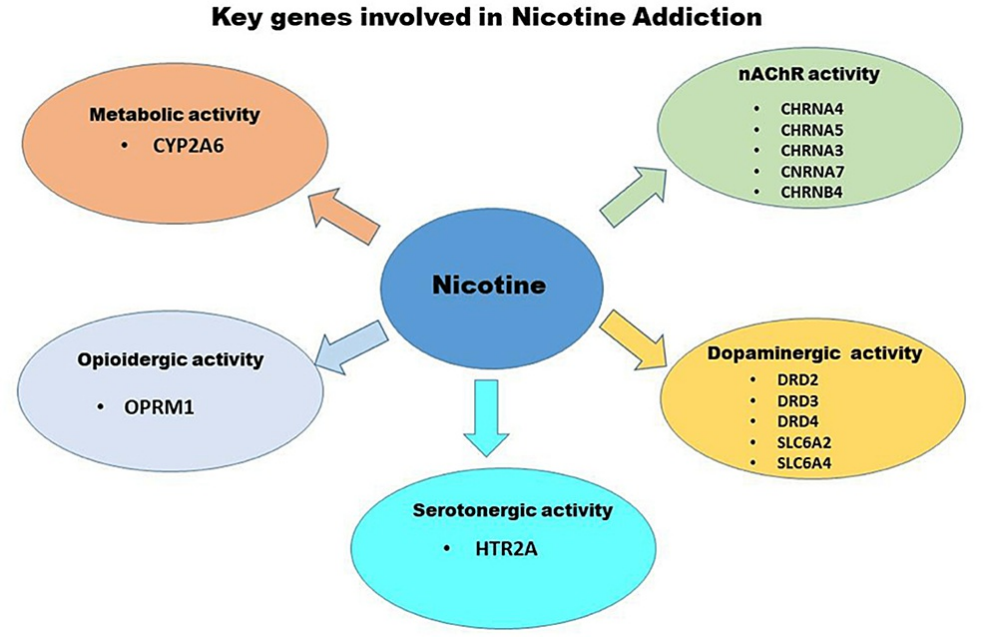
Neurotransmitters and genetic interplay in nicotine addiction

Nicotine is a sympathomimetic drug that causes the release of catecholamines and increases heart rate, cardiac contractility, constricts cutaneous and coronary blood vessels and increases blood pressure [8]. A study done by Mahajan et al. depicts the interplay between neurotransmitters and genes involved in nicotine addiction as shown in Figure 2.

Nicotine has toxic effects on the endothelium, and thus nicotine may play a key role in impaired nitric oxide synthase-dependent vasoreactivity observed in tobacco users. Nicotine produces morphologic abnormalities of the endothelium, causing direct alteration of the vascular reactivity. It makes its toxic effect via the production of oxygen-free radicals.

A study done in Asia found that smokers had a higher incidence of heart failure than former and never smokers. This aligns with previous studies reporting that smoking negatively affects cardiac physiology. The mechanism involves endothelial dysfunction by reducing nitrogen monoxide production, pro-thrombotic conditions, and activating inflammatory routes. These factors and the increased amounts of coronary atherosclerosis may be responsible for the increased risk of hypertension, coronary heart disease, and atrial fibrillation, potentially contributing to the association of current smokers with higher a heart failure incidence [9].

A multicenter study suggested that smokers with rheumatoid arthritis require more aggressive therapy with disease-modifying anti-rheumatic drugs. In addition, they have a poorer response to anti-tumor necrosis factor treatment and have an increased risk of cardiovascular disease. The mechanisms remain poorly defined. However, systemic inflammation and well-known cardiovascular disease risk factors, such as smoking and high cholesterol levels, are possible culprits. All this indicates that smokers with rheumatoid arthritis could benefit from smoking cessation regarding their anti-rheumatic medical treatment [10].

**Figure 3:**
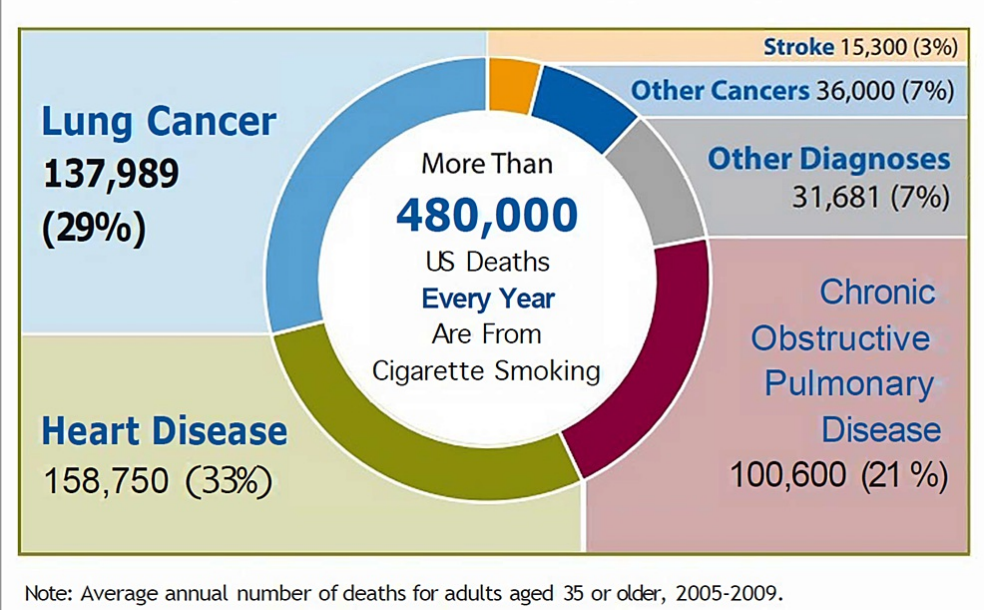
Annual number of deaths from cigarette smoking

Cigarette smoking reduction has also been associated with a remarkable decrease in the risk of lung cancer for participants, reducing the number of cigarettes smoked per day by 50 percent. However, there were mixed results for the same variable for cardiovascular conditions such as stroke and coronary artery disease [10]. The 2014 general surgeon's report depicts the average annual mortality for adults greater 35 years between 2005 and 2009 in Figure 3.

Guidelines to quit smoking

The Royal College of Physicians investigated clinical guidelines and recommendations issued by UK National or European Transnational Medical Specialty Associations and Societies issued between 2000 and 2012 on various diseases caused by smoking. Worthy of note, 60% of the guidelines mentioned smoking as a disease-causing risk factor. In contrast, 40% recommended smoking cessation. Only 19% included and provided detailed information on the process and steps to smoking cessation in the final draft of the guidelines and recommendations [11].

A review of Cochrane studies on pharmacological intervention for tobacco cessation found that nicotine replacement therapy (NRT), bupropion, and varenicline are more effective for quitting tobacco use as compared to placebo. Varenicline increased the odds of tobacco cessation compared to other forms of pharmacological intervention studied and is more effective than a single form of NRT and bupropion. Also, nortriptyline, bupropion, and clonidine have been used for tobacco cessation, but their extensive side effects, including cardiac conduction abnormality, seizure, and hypotension, have limited their use [1].

Further studies on the nicotine receptor partial agonists for smoking cessation in which Cytisine showed increased chances of quitting, although absolute quit rates were modest in two recent trials. Varenicline at a standard dose increased the chances of successful long-term smoking cessation between two- and three-fold when compared with pharmacologically unassisted quit attempts. Nausea was reported as the most common side effect of Varenicline [12].

Exercise interventions for smoking cessation have been explored. There is no significant evidence or benefit in adding either resistance or cardiovascular exercise to smoking cessation intervention to promote and improve abstinence [13]. Worthy of note are nursing interventions for smoking cessation in the form of advice/counseling during healthcare visits. This was found to be reasonably effective, especially if done for an extended period [14].

Impact of discontinuing smoking and benefits

Tobacco smoking is the single greatest preventable cause of mortality [15]. Smoking is a recognized risk factor for cardiovascular disease, and the Framingham Heart Study identified the relationship between smoking and cardiovascular disease in men [16].

At least one decade of life is lost by current active smokers compared to those who have never smoked [17]. Jeong et al. compared the impact of smoking cessation versus the reduction in the number of cigarettes smoked on cardiovascular health and only found a reduced risk of stroke and myocardial infarction in the quitter group [18]. Quitting smoking earlier in life has been shown to confer better survival benefits than quitting later. Jha et al. compared the impact of smoking cessation among different age groups; smokers who quit at 25 to 34 years had survival curves nearly identical to those who never smoked, with a ten-year survival gain compared to current smokers. Survival curves (hazard ratio, 1.2) were somewhat worse for smokers who quit at 35 to 44 years of age (median, 39) than those who had never smoked. However, cessation at about 39 years reduced the excess risk of death from any cause by 90%, with nine years gained compared to current smokers. Those that quit between 55 and 64 years of age gained about four years of life compared to current smokers [19].

Smoking cessation does not immediately reduce the risk of cardiovascular mortality. An observational cohort study of 8,770 participants by Duncan et al. among heavy smokers (20 pack-years) demonstrated smoking cessation was associated with a significantly lower risk of cardiovascular disease within five years relative to current smokers. However, relative to never smokers, the cardiovascular disease risk of former smokers remained significantly elevated beyond five years after smoking cessation. This study established that smoking cessation is beneficial, but cardiovascular disease risk from smoking slowly declined over decades [20]. Weight gain following smoking cessation can occur in some individuals 22. Sahle et al., in a recent cohort study, found that those who quit smoking had a greater increase in weight (mean difference [MD], 3.14 kg; 95% CI, 1.39-4.87) and BMI (MD, 0.82; 95% CI, 0.21-1.44) than continuing smokers [21].

Yang et al. found an increased risk for type 2 diabetes among recent quitters than current smokers, (hazard ratio, 1.22; 95% confidence interval [CI], 1.12 to 1.32). The risk peaked within 5 to 7 years after quitting and gradually decreased. This risk was also directly proportional to the weight gain and was not increased in quitters without weight gain [22]. Post-smoking cessation weight gain and increased risk for type II diabetes are common concerns for smokers contemplating quitting. Xiaowen et al., in a meta-analysis, examined post-cessation weight gain and CVD and found that smoking cessation was associated with a significantly lower risk of CVD and all-cause mortality regardless of post-cessation weight gain [23]. Dehghani reported that participants who quit smoking entirely after acute coronary syndrome gained weight compared to those who partially or never quit groups.

Also, arterial stiffness is a known marker for increased risk of hypertension, a meta-analysis by Saz-Lara et al. evaluated the effect of smoking cessation on arterial stiffness, and the studies showed that arterial stiffness decreased following smoking cessation 10. Studies have shown that concerning the incidence of heart failure, non-smokers have a reduced chance of heart failure compared to their counterparts who are currently smoking or are former smokers [24]. A meta-analysis carried out in China on the impact of smoking on early complications after liver transplantation found that cigarette smoking was not associated with higher comprehensive complication intervention scores, and smokers did not have a higher risk for developing acute kidney injury, hepatic artery thrombosis, and biliary complications, after liver transplantation [24].

As regards to behavioral and mental health, it has been found that sustained tobacco abstinence for about 52 weeks reduced the 10-year cardiovascular risk in outpatients with serious mental illness. However, these participants had significant post-cessation weight gain with a high prevalence of obesity, diabetes, and hypertension. Chances of early and premature menopause have been markedly associated with the duration, intensity, cumulative dose, and time of smoking initiation in the studied population. There was tremendous benefit noted for women who quit smoking from experiencing early or premature menopause [25].

Recommendations and future trends

In the CDC's analysis of the Surgeon General's report on smoking in 2020, the percentage of former smokers over 18 who quit smoking according to age group was highest in the group >65 years and lowest in the group 18-25 years [26]. It was reported that as of 2020, more than three of every five smokers had quit smoking. This is largely due to increased advice from health professionals on smoking cessation as part of routine conversations with their patients [26]. However, this trend has marked disparities as some subcategories of the population are still riddled with a high burden of cigarette smoking and its ripple effect [26].

The future trend should be affixed on reaching the young population, particularly in the age 18-24 years category, as this can be done early, reversibly, and with the most benefit and success [26]. Several pieces of evidence have postulated that increasing the price of tobacco cigarettes may reduce the prevalence of smoking and propel cessation. Other policies include expanding smoke-free zones in public places, expanding the media campaign against tobacco smoking in all school institutions and levels and instituting comprehensive tobacco control programs [26].

## Conclusions

Smoking remains an independent risk factor for multiple cardiovascular diseases. Smoking cessation has the propensity to mitigate cardiovascular diseases and complications especially when achieved on a timely scale. Smoking cessation would reduce overall morbidity and mortality. The general trend over the last decade has seen a significant drop in tobacco use, although there may be a concern for the increased use of vaping/smokeless cigarettes in young adults. More awareness especially in the younger population to prevent initial tobacco use and increase the incorporation of tobacco cessation protocols in the healthcare institution settings for older patients with tobacco use disorder and other chronic diseases is warranted.
